# Termination of seizures by ictal transcranial focal cortex stimulation

**DOI:** 10.1002/epi4.12728

**Published:** 2023-03-26

**Authors:** Martin Hirsch, Volker Arnd Coenen, Andreas Schulze‐Bonhage

**Affiliations:** ^1^ Faculty of Medicine, Epilepsy Center University of Freiburg Freiburg Germany; ^2^ Department of Stereotactic and Functional Neurosurgery Faculty of Medicine, University of Freiburg Freiburg Germany; ^3^ Neuromodule Basics University of Freiburg Freiburg Germany; ^4^ Center of Deep Brain Stimulation University of Freiburg Freiburg Germany

**Keywords:** ictal stimulation, neurostimulation, seizure termination, transcranial stimulation

## Abstract

Whereas high‐level evidence exists on chronic neuromodulatory effects of different brain stimulation approaches in reducing seizure frequency, evidence for acute antiseizure effects of electrical brain stimulation during seizures is sparse. As part of an ongoing trial, we implanted a patient with a novel focal cortex stimulation (FCS) device with a Laplacian electrode placed over a precentral focal cortical dysplasia. The baseline seizure frequency was 125 per month, consisting of (i) focal aware sensory seizures that invariably progressed to uni‐ or bilateral tonic contraction and clonic jerking, and (ii) primary motor seizures. Besides an overall reduction in seizure frequency, on‐demand stimulation had an immediate effect on seizures with a sensory phase, whereby 63%‐86% of these seizures were terminated by ictal stimulation. These observations provide the first evidence that ictal self‐triggered transcranial focal cortex stimulation can significantly interfere with the progression of seizure semiology.

## 1 INTRODUCTION

Three implantable neurostimulation systems have been approved thus far for the treatment of epilepsy, vagus nerve stimulation (VNS),^1^ deep brain stimulation of the anterior thalamic nuclei (DBS),^2^, ^3^ and responsive neurostimulation (RNS) of the epileptic focus.^4^

Although there is high‐level evidence that various forms of brain stimulation can exert chronic neuromodulatory effects that lead to a reduced seizure frequency, evidence for acute seizure termination of electrical brain stimulation during seizures is very limited^5^, ^6^, ^7^, ^8^ even for devices designed to apply stimulation also during seizures (RNS or patient‐triggered and heart‐rate‐sensor‐based VNS).^9^, ^10^, ^11^ Transcranial focal cortex stimulation (FCS) is a novel treatment option that is presently being evaluated in a clinical trial for the treatment of pharmacoresistant focal epilepsy.^12^ The therapy consists of continuous interictal stimulation and ictal stimulation triggered by patients upon their own perception of early seizure symptoms. Here, we report for the first time on seizure termination effects of patient‐triggered transcranial focus stimulation in one patient.

## 2 CASE PRESENTATION

**FIGURE 1:**
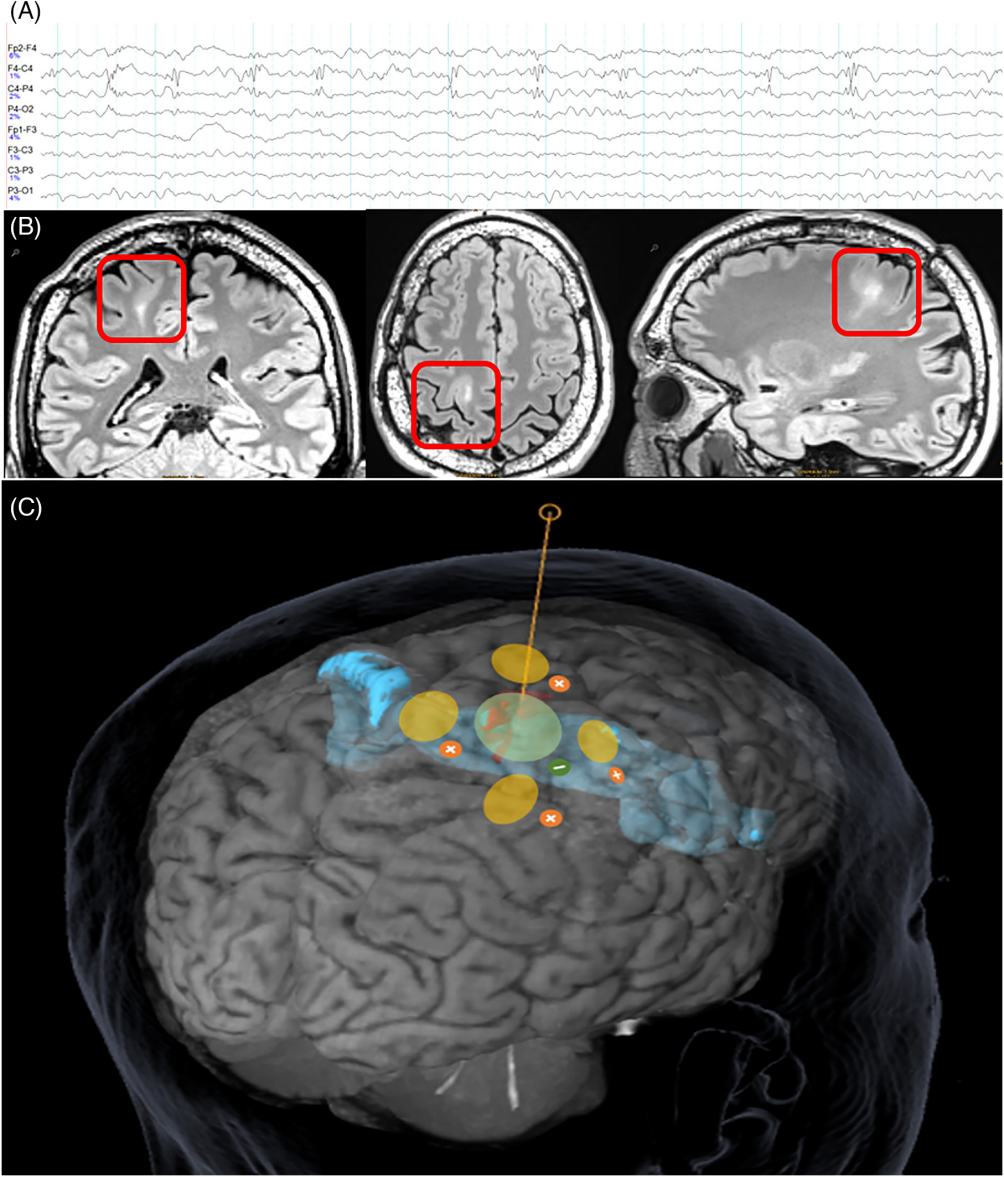
Electrode positioning based on delineation of the epileptic focus by EEG and MRI. A, Scalp EEG with frequent epileptic polyspike discharges in EEG over the right central region. B, MR Imaging of a right premotor suspected bottom‐of‐sulcus dysplasia with a typical tail sign (Siemens PRISMA 3T, 3D T2‐FLAIR‐MR images in coronal, axial, and sagittal direction). C, positioning of the electrode array with electrodes in green and yellow over the bottom‐of‐sulcus dysplasia (in red) and in relation to the primary motor cortex (blue segment).

A 44‐year‐old male patient was enrolled in the PIMIDES‐I clinical trial based on: (i) the diagnosis of pharmacoresistant focal epilepsy, which first manifested at the age of 6 and is attributed to a neocortical epileptogenic lesion at the dorsolateral convexity of the brain, and (ii) his ability to initiate bolus stimulation during the focal aware phase of his seizures. At the time of enrolment, the patient had prolonged focal aware sensory seizures that lasted several minutes and regularly progressed to focal motor seizures, often resulting in postural instability and falls. Based on his refractoriness to 13 previously administered antiseizure medications, the patient underwent both non‐invasive and invasive presurgical monitoring. Scalp EEG recordings showed polyspike discharges over the right precentral region, concordant with MR imaging suggestive of a bottom‐of‐sulcus type II focal cortical dysplasia (Figure 1). Subdural grid recordings showed long‐lasting, low‐amplitude fast activity in the seizure onset zone, which then progressed to rhythmic beta activity over extended areas upon the onset of motor symptoms. However, due to an overlap between the ictal onset zone and the primary motor representation of the left hand, resective surgery could not be offered as a therapeutic option.

For the purposes of PIMIDES‐I trial, a five‐channel electrode array (Figure 1) was implanted on the area of the skull overlying the patient's precentral focal cortical dysplasia. Stimulation was initiated 1 month postimplantation, with continuous stimulation consisting of a combination of interictal DC‐like stimulation (DLS) for 20 minutes and intermittent high‐frequency stimulation (HFS) at 100 Hz. In addition, patient‐triggered bolus stimulation was enabled using a handheld device (30 seconds duration, 4 mA, 100 Hz, 160 μs pulse width). Seizure types and frequency were documented by the patient in a seizure diary. The antiseizure medication regimen of the patient remained unchanged during the entire reporting period.

Prior to activation of neurostimulation, the patient documented focal sensory seizures with numbness and a feeling of tension in his left shoulder region, gradually spreading to the left arm and hand, and invariably progressing to a focal motor seizure with tonic contraction of the left extremities; this occasionally also involved tonic contraction of the right side and clonic jerking of the left arm. Both awareness and responsiveness were preserved, but the ictal motor phenomena led to falls, prompting the patient to wear a helmet to protect himself from head injuries. Prior to stimulation, the patient reported a mean seizure frequency of 5 per day. Whereas seizures arising during reduced vigilance were reported as motor only, those arising from wakefulness showed a strict evolution of symptoms from sensory to motor, with no isolated sensory seizures having occurred for several years; the initial sensory phase of the seizures would last up to 10 minutes. Of note, on‐demand stimulation could only be initiated during seizures occurring during wakefulness.

**FIGURE 2:**
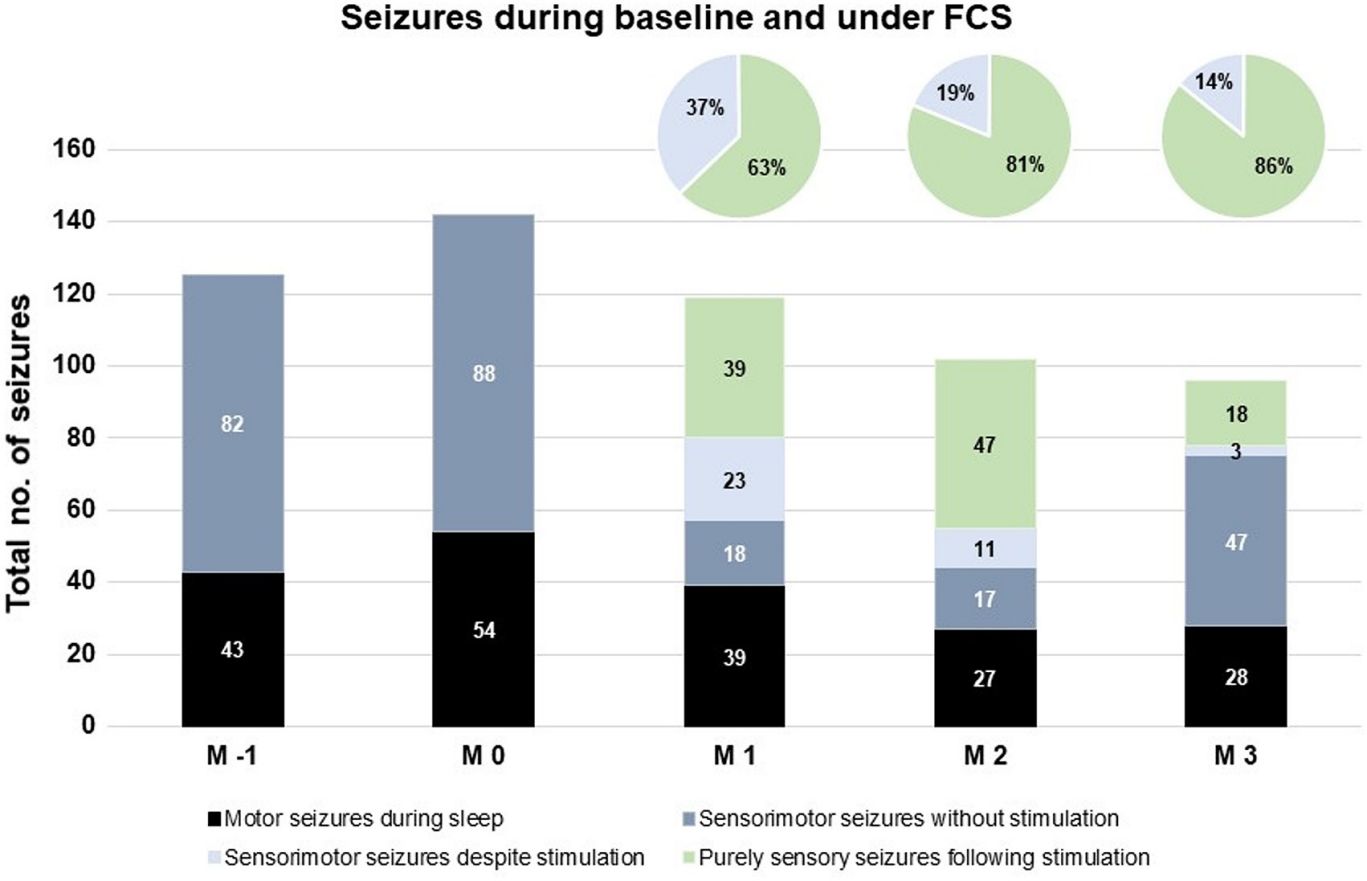
Monthly seizure counts in the case study patient preceding device implantation (M‐1), after device implantation (M0) and during active stimulation (M1‐M3). Absolute numbers are given in the bar diagram, percentages of aborted vs. continuing seizures with an initial sensory‐aware phase in the circle diagrams. Abortion of seizures during the sensory‐aware phase following patient‐triggered ictal focal cortex stimulation during M 1‐3 occurred in 63%/81%/86% of seizures in which stimulation was performed. Furthermore, there was a gradual reduction in the total seizure frequency over the 3‐month stimulation period by 23% in M3 of stimulation compared with the prospective baseline (M‐1).

Figure 2 displays effects of stimulation on seizure frequency and semiology during the observation period. Aside from an overall reduction in seizure frequency, patient‐triggered bolus stimulation during the sensory phase of his seizures prevented the evolution to the motor phase and terminated seizures in the majority of cases. All seizures arising from wakefulness without ictal stimulation continued to progress from the initial sensory to the motor phase. The efficacy of ictal stimulation in suppressing the progression of the seizures during the sensory phase was 63% in the first month of stimulation and increased to 86% by the third month. In contrast, motor seizures from sleep that occurred without ictal stimulation remained unchanged in semiology, although they did occur at a lower rate.

This case study shows that patient‐triggered ictal high‐frequency stimulation of the epileptic focus during the initial focal aware phase can largely prevent further seizure progression, as well as the evolution of seizure semiology. Prior to focal cortex stimulation, 100% of the patient's focal sensory seizures proceeded to the manifestation of motor symptoms, whereas such progression only occurred in 14%‐37% of seizures when ictal focus stimulation was applied.

The pronounced effects of ictal transcranial focal cortex stimulation observed here are of general interest in the framework of neurostimulation in epilepsy. Both vagus nerve stimulation (VNS) and thalamic deep brain stimulation (DBS) are thought to induce network neuromodulation by way of intermittent interictal effects at sites distant to the epileptic focus. In this settings, the efficacy of stimulation increases over periods of several months to years when identical stimuli are applied, suggesting a chronic neuromodulatory rather than an acute effect on seizures. Similarly, in several experimental models in vivo, it has been reported that there is a lack of stimulation efficacy once an ictal pattern has been already initiated, while closed‐loop application of ictal VNS has no influence on ictal EEG discharges and behavioral seizure manifestations, in contrast to efficacy of pre‐ictal stimulation. In a trial on tachycardia‐triggered vagus nerve stimulation, progression to tonic–clonic seizures was not aborted by ictal VNS, neither was the duration of seizures with objective signs shortened.^13^ Nonetheless, ictal stimulation has been offered as a feature of VNS, initially as patient‐induced device activation and more recently as automated triggering based on tachycardia detection.^14^ There are, however, no data available that clearly distinguish between the efficacy of these different forms of stimulation, with the exception of one 2015 case study reporting an association between shorter seizure duration and ictal stimulation.^5^

Acute suppressive effects of direct cortical stimulation on ictal electrographic patterns have been reported for intraoperative stimulation‐evoked seizures^15^ as well as for after discharges arising from extraoperative cortical stimulation.^16^, ^17^ In experimental models, absences have been successfully terminated by EEG‐triggered stimulation.^18^ For efficacy of closed‐loop ictal stimulation in the setting of spontaneously arising focal seizures, however, the evidence is sparse, although a device offering intracranial responsive neurostimulation for the treatment of focal epilepsy was intended to exert its effects by early detection and stimulation of ictal electrographic discharges. Given the gradual increase in efficacy over time^4^, ^10^, ^19^ and the several thousand bursts of stimulation performed on average per day,^4^, ^10^ distinguishing between neuromodulatory versus ictal effects is difficult, and efficacy has even been shown in cases in which acute effects on seizure semiology were absent. Closer electrophysiological investigations showed a lack of correlation between direct/short‐term electrical effects of responsive neurostimulation of the focus, whereas indirect modulatory effects like a fragmentation of electrographic discharges were positively correlated to treatment efficacy of responsive focus stimulation.^11^

Timely targeted neurostimulation to control severe seizures is of high interest in epilepsy, given that seizures occupy <0.1% of time. Evidence for the ictal effects of neurostimulation is thus of particular interest. We here provide the first evidence that epicranial focal cortex stimulation with high‐frequency pulses can acutely alter brain dynamics in the cortical epileptogenic zone, and terminate sensory seizures prohibiting their otherwise invariable progression to the recruitment of motor regions with severe motor manifestations. The development of closed‐loop approaches for ictal electrical cortex stimulation with a minimally invasive device as used here may thus fill a therapeutic gap for patients with epilepsy. In our experience from the clinical trial recruitment process at our tertiary epilepsy center with a pre‐selected patient population, around 10% of patients could be potential candidates for FCS. In half of these patients, the duration of the focal aware phase of their seizure and the responsiveness of the patients are sufficient to derive a potential benefit from on ictal self‐triggered FCS.

## 3 METHODS

### 3.1 Study registration and funding

The study is registered under DRKS00017833 and CRD42021266440.

The study was supported by grant of the German Federal Ministry of Education and Research (13GW0269B) and sponsored by the company PRECISIS GmbH (Heidelberg, Germany). The study was approved by the local ethics committee.

### 3.2 Participant

A 44‐year‐old man with a childhood‐onset severe epilepsy highly refractory to multiple antiseizure medications was enrolled in the PIMIDES‐I clinical trial. The patient gave written informed consent for participation in a clinical trial. Key inclusion criteria included neocortical focus localization and accessibility of the epileptic focus for stimulation via an epicranially placed electrode, and the patient's ability to initiate a stimulation bolus during the focal aware phase of seizures.

### 3.3 Clinical measures

Seizure types, seizure occurrence, and presence or absence of ictal stimulation were documented by the patient in a study‐specific seizure diary.

### 3.4 Neurostimulation

DC‐like stimulation mode (DLS) consisted of 20 ms cathodal pulses at an intensity of 2 mA with an equilibration pulse of 100 ms duration at 0.4 mA in daily 20 minutes sessions for long‐term neuromodulation. Alternating current (AC) high‐frequency (100 Hz) stimulation mode was carried out intermittently by applying short bursts every 2 minutes (single pulse duration 160 μs, burst duration of 500 ms, intensity 4 mA). Patient‐triggered bolus stimulation was carried out with a 160 μs pulse width, 100 Hz bursts applied for 10‐60 seconds using an external EASEE® Access handheld device. The duration of the stimulation bolus was fixed and the patient had no possibility to change it.

All stimulation forms were below perception threshold.

## AUTHOR CONTRIBUTIONS

MH and ASB contributed to the acquisition and analysis of data. MH, ASB, and VC contributed to the drafting of the text and preparing of figures.

## CONFLICT OF INTEREST STATEMENT

ASB has received research support for leading the clinical trial and recruiting patients at the Freiburg Epilepsy Center from the company PRECISIS producing the EASEE device, from the German Ministry of Research and Education, and from the Ministry of Science Baden‐Wuerttemberg for projects to develop intelligent implants for neurostimulation. MH was an investigator in the clinical trials EASEE and PIMIDES and recruited patients at the Freiburg Epilepsy Center.
